# Renal Transplant Recipients Treated with Calcineurin-Inhibitors Lack Circulating Immature Transitional CD19^+^CD24^hi^CD38^hi^ Regulatory B-Lymphocytes

**DOI:** 10.1371/journal.pone.0153170

**Published:** 2016-04-05

**Authors:** Bastian Tebbe, Benjamin Wilde, Zeng Ye, Junyu Wang, Xinning Wang, Fu Jian, Sebastian Dolff, Manfred Schedlowski, Peter F. Hoyer, Andreas Kribben, Oliver Witzke, André Hoerning

**Affiliations:** 1 Department of Nephrology, University Hospital Essen, University Duisburg-Essen, Essen, Germany; 2 Department of Pediatrics II, Pediatric Nephrology, Gastroenterology, Endocrinology and Transplant Medicine, Children’s Hospital Essen, University Duisburg-Essen, Essen, Germany; 3 Institute of Medical Psychology and Behavioral Immunobiology, University Hospital Essen, University of Duisburg-Essen, Essen, Germany; 4 Department of Infectious Diseases, University Hospital Essen, University Duisburg-Essen, Essen, Germany; 5 Department of Pediatrics and Adolescent Medicine, University Hospital Erlangen, Friedrich-Alexander University of Erlangen-Nuremberg, Erlangen, Germany; University of Toledo, UNITED STATES

## Abstract

**Background:**

CD19^+^CD24^hi^CD38^hi^ transitional immature B-lymphocytes have been demonstrated to play an important role in regulating the alloimmune response in transplant recipients. Here, we analyzed the effect of calcineurin inhibition on these peripherally circulating regulatory B-cells (Breg) in renal transplant recipients receiving cyclosporine A (CsA) or tacrolimus.

**Methods:**

PBMCs from healthy subjects (HS) (n = 16) and renal transplant recipients (n = 46) were isolated. Flow cytometry was performed for CD19, CD24, CD38 and IL-10 either after isolation or after 72 hours of co-culture in presence of PMA/Ionomycin and TLR9-ligand in presence or absence of increasing concentrations of tacrolimus or CsA.

**Results:**

The amount of CD19^+^ B-cells among lymphocytes was ∼9.1% in HS, ∼3.6% in CsA (n = 11, *p*<0.05) and ∼6.4% in TAC (n = 35, *p*<0.05) treated patients. Among B-cells, a distinct subset of Breg was found to be 4.7% in HS, 1.4% in tacrolimus treated patients and almost blunted in patients receiving CsA. Similarily, ∼4% of B-cells in HS and even fewer in CsA or tacrolimus treated patients produced IL-10 (0.5% and 1.5%, *p*<0.05) and this was confirmed both in non-transplanted CsA-treated healthy subjects and in *in vitro* co-culture experiments. Among 29 patients with <1% of Breg, 9 cases (31%) displayed an allograft rejection in contrast to only one case of rejection (6%) among 17 patients with >1%.

**Conclusion:**

Calcineurin inhibitors reduce number and IL-10 production of Bregs in the peripheral circulation of both renal transplant recipients and non-transplanted healthy subjects. CNI induced Breg reduction is not restricted to a solid organ transplant setting and is not mediated by co-medication with steroids or MPA. A low proportion of Breg cells is associated with an elevated frequency of allograft rejection events.

## 1 Introduction

B-cells shape the humoral immunity and are classically considered to amplify the immune response because of their capability to produce antibodies (including autoantibodies) as well as acting as antigen-presenting cells to modulate T-cell-mediated immune responses [1, 2]. After solid organ transplantation, the production of donor-specific allo-antibodies (DSA) is involved in both acute and chronic allograft rejection [3, 4]. By contrast, immature subsets of B-cells termed regulatory B-cells have recently been shown in mice and humans to mediate protective immune responses by producing regulatory cytokines such as IL-10, TGF-b, IL-35 and directly interacting with pathogenic T-cells via cell-to-cell contact [1, 2, 5–9]. The Breg cell population appears to be heterogenous as different murine B-cell subsets such as CD1d^hi^CD5^+^ [10] and Tim-1^+^IL-10^+^ B-cells [11, 12] have been described to exert immunoregulatory function. In humans, the identification of B-cells with regulatory properties has first been demonstrated in several studies in allergic [13] and autoimmune diseases such as systemic lupus erythematosus [14] or gianT-cell arteritis [15]. An exact definition of human Breg cells by lineage-specific surface markers is lacking [9, 16]. Studies in patients with relapsing-remitting multiple sclerosis or type 1 diabetes demonstrated that *ex vivo*-activated B-cells produced less IL-10 than B-cells from healthy subjects, suggesting that insufficient IL-10 secretion by B-cells might facilitate autoimmune pathogenesis in humans [17, 18]. A widely accepted phenotypic characterization of Bregs in humans was suggested by Blair et al. [14]. In their studies, the immature transitional CD19^+^CD24^hi^CD38^hi^ B-cell subset was shown to enrich for IL-10+ Bregs and these cells suppressed CD3-mediated activation and differentation of Th1-cells via both secretion of IL-10 and cell-cell interaction [14]. In addition, Tedder and colleagues [19] identified a CD19^+^CD24^hi^CD27^+^ memory B-cell subset harboring a similar number of IL-10 producing cells that have been identfied to inhibit TNFα production of CD4^+^ Th-cells and monocytes.

In transplant immunology, recent findings from both experimental murine models and human studies pointed out that distinct B-cell subsets exert an immunoregulatory activity and may both participate in maintaining long-term allograft function and contribute in preventing allograft rejection [20]. This is a rather surprising finding because the presence of donor-specific allo-antibodies in transplant recipients is often associated with a decreased allograft function. Recent microarray analysis and Real Time PCR studies in operational tolerant allograft recipients (stable allograft function one year after withdrawal of immunosuppression) identified a B-cell specific gene signature indicating that subpopulations of B-cells may indeed play a relevant role in tolerance induction after solid organ transplantation [21, 22]. Such a tolerogenic signature was characterized by an increase in immature transitional CD19^+^CD24^hi^CD38^hi^ B-cells exhibiting potent immunoregulatory properties compared to recipients with stable allograft function or with biopsy-proven chronic rejection. Although much attention has been focused on regulatory B-cell subsets in human and murine models of autoimmunity, the understanding of their role in solid organ transplantation is still limited. Moreover, information on the effect of immunosuppressive drugs on occurence and function of regulatory B-cells in renal transplant recipients receiving a calcineurin inhibitor based immunosuppressive therapy is scarce.

In this study, we analyzed the distinct subset of CD24^hi^CD38^hi^ Breg cells in renal transplant recipients receiving a calcineurin inhibitor based immunosuppression and compared these results with healthy subjects. The main finding was that calcineurin inhibition reduced the amount of CD24^hi^CD38^hi^ Bregs and inhibited their IL-10 production independent of additional co-medications (e.g. steroids or mycophenolate derivatives) and that a decrease of peripherally circulating Bregs seems to be associated with the incidence of allograft rejection events.

## 2 Patients, Materials and Methods

### Material and Methods

#### Renal transplant patients

Fourty-six Caucasian patients (26 male, mean age 54 ±14 years, range 23–74) were included that underwent renal transplantation receiving a CNI in combination with mycophenolate acid derivatives and steroids (TAC n = 35; CsA n = 11). None of these patients underwent kidney transplantation before. Mean trough level at timepoint of sample assessment was for TAC 6.2±2.7 ng/mL and 106±30 ng/mL for CsA. Except four, all patients underwent glucocorticoid therapy, mean dosage of prednisone was 6.6±3.3 mg/day. Mycophenolate mofetil (MMF) was given in a fixed dose of one to two times 1 g/day (n = 22) and mycophenolate sodium (MPA one to two times 720 mg/d (n = 14). None of the patients received a B-cell depleting therapy.

Heparinized whole blood samples were collected in the outpatient clinic at periodical routine follow up visits starting six weeks after transplantation. Clinical chemistry was assessed out of the same samples that were used for FACS aquisition. Patients showing signs of acute infections (CrP >0.5 mg/dL) or a history of autoimmune or malignant diseases were excluded from analysis. The mean time after transplantation at the timepoint of sample assessment by flow cytometry was 41 ±45 months. Twelve living donations were among the investigated patients. Ten patients developed a biopsy proved rejection within 24 months before or after measurement. After rejection, flow cytometry analysis was performed earliest after two months. Blood samples of patients with renal allograft rejection were assessed two months after the high pulse dosage steroid treatment was finished. Allograft function and the glomerular filtration rate (GFR) was estimated and calculated using the MDRD formula (ml/min). Informed consent was obtained before entering the study. The protocols for the investigations in patients and healthy subjects was approved by the ethics committee of the Medical Faculty of the University Duisburg-Essen (05–2803). None of the transplant donors were from a vulnerable population and all donors or next of kin provided written informed consent that was freely given.

#### Healthy subjects

Sixteen healthy subjects served as a control group. There was no significant difference in age or sex compared to the patient groups. Blood specimen of four healthy subjects treated with CsA were kindly provided by the group of Professor M. Schedlowski (Institute of Medical Psychology and Behavioral Immunobiology, University Hospital Essen, University of Duisburg-Essen, Essen, Germany).

#### Antibodies and reagents

Mouse anti-human CD19-PerCP or APC (HIB19), mouse anti-human CD24-FITC (eBioSN3), mouse anti-human CD38-APC (HIT2) and corresponding Ig-subclass-specific isotype controls were purchased from eBioscience (Frankfurt, Germany). Mouse anti-human CD25-PE (M-A251), mouse anti-human CD4-PerCP (L200), mouse anti-human CD127-FITC (hIL-7R-M21) and corresponding isotype controls were purchased from BD Biosciences (San Jose, CA, USA). For intracellular staining rat anti-human IL-10-APC, PE (JES3-9D7) from eBioscience (Frankfurt, Germany), mouse anti-human IFN-γ-APC (B27) from BD Biosciences and appropriate isotype controls were used. The cell stimulation cocktail plus protein transport inhibitor 500x was purchased from eBioscience, CpG ODN2006 from Invivogen (San Diego, USA). CsA was purchased from Novartis (Basel, Switzerland) and tacrolimus from Sigma-Aldrich (Hamburg, Germany).

#### Surface staining

**Fig 1:**
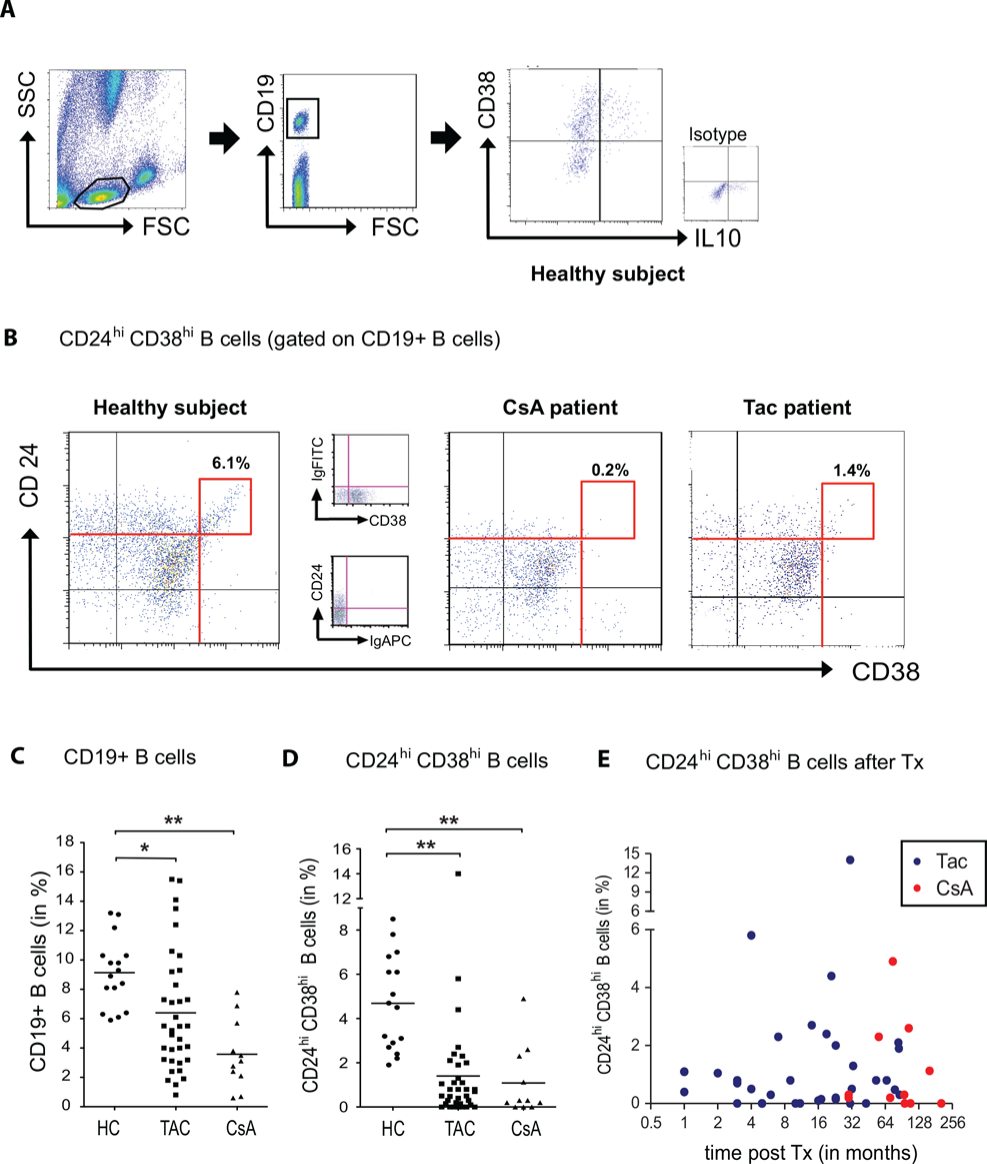
Calcineurin inhibitors reduce the expression of CD24 and CD38 on CD19^+^ B lymphocytes. The expression of CD24, CD38 and IL-10 after gating on CD19^+^ B-cells is shown in a representative healthy subject, indicating that only a minority of IL-10 producing B-cells highly express CD38 **(A)**. The dot blots in **B** depict CD24^hi^CD38^hi^ B-cells (red-framed boxes) in a representative healthy subject, a renal transplant recipient receiving a CsA or tacrolimus based immunosuppression. The respective isotype control staining is depicted in between. The scatter plot graphs in **C** and **D** summarize the results and show that treatment with tacrolimus (n = 35) or CsA (n = 11) not only reduce the percentage of peripherally circulating B-lymphocytes **(C)** but also affect the CD24^hi^CD38^hi^ B-cell subset **(D)**. In contrast to healthy subjects (n = 16), the CD24^hi^38^hi^ expressing B-cell subset of renal transplant patients receiving a calcineurin inhibitor were significantly reduced or even blunted **(B, D)**. No correlation was found between the amount of CD24^hi^CD38^hi^ B-cells and time of sample assessment after transplantation **(E**, Spearman test, *r* = -0,01, *p* = 0,9450).

Heparinized venous blood was collected from all patients. 100μl was immediately used for surface staining using antibodies against CD19, CD24 and CD38. After 20 minutes of incubation at room temperature in the dark, red blood cells were lysed following the manufacturer´s protocol (PeliLyse buffer A1 set, Sanquin, Amsterdam, The Netherlands). Four color FACS analyses was performed by gating first on the lymphocyte and subsequently on the CD19^+^ B-cell population. Gating for CD19^+^CD24^hi^CD38^hi^ cells was subsequently constantly performed as shown in Fig 1. Aquisition was performed on a FACSCalibur (BD Biosciences) using CellQuest software (BD Biosciences). Data were analyzed using FlowJo Software, Version 8.7.3 (Tree Star, Inc., Ashland, USA).

#### Cell culture and stimulation

Peripheral mononuclear blood cells (PBMCs) were isolated by Ficoll density gradient centrifugation immediately after collecting the blood samples. Isolated PBMCs were washed, counted and incubated at a concentration of 1x10^6^ cells/mL in 12-well plates at 37°C and 5% CO_2_. Isolated B-cells were cultured in 96-well flat bottom plates. Cell culture was performed in complete RPMI-1640 media containing 10% fetal calf serum, 2mM L-glutamine, 100 U/ml penicillin/streptomycin (1%, all from Invitrogen), 1% sodium bicarbonate and 1% sodium pyruvate (Cambrex). PBMCs were stimulated with 1μM ODN2006 Type B CPG (Invivogen, San Diego, USA) per well or were left unstimulated for control purposes and incubated for 72 hours. For the last six hours 2μl/mL cell stimulation cocktail plus protein transport inhibitors 500x containing PMA (40.5μM), Ionomycin (670μM), the protein transport inhibitors Brefeldin A (5.3mM), and Monensin (1mM) was added. Viability of cells before and after cell culture was assessed using 7-AAD staining (eBioscience) and methylen-blue.

#### *In vitro* cell culture assay

PBMCs were isolated and stimulated as described before. For some experiments, B-cells were isolated from buffy coats of healthy blood donors by negative selection using a magnetic column based system B-cell isolation kit II (Miltenyi Biotec, Bergisch Gladbach, Germany). Purity was typically above 95%. Incubation was performed in presence or absence of TAC or CsA. Increasing concentrations of tacrolimus (1, 5, 10 ng/mL) and CsA (1, 10, 100 ng/mL) were used according to clinical established trough levels.

#### Intracellular staining

After stimulation and 72 hours of cell culture PBMCs were washed, stained for CD19, CD24, CD38 and incubated for 20 minutes at room temperature in the dark. Then intracellular staining according to manufacturer’s protocol for IL-10 and IFN-γ was performed using Fix&Perm cell permeabilisation kit (Invitrogen, Karlsruhe, Germany). After staining and washing, FACS analyses were performed immediately.

#### Statistical analysis

Statistical analyses were carried out using Graph Pad Prism 5.0 (Graph Pad Software, La Jolla, USA). If not mentioned otherwise, values are given as mean ± standard deviation. Results were compared using the Mann-Whitney U test, correlation analysis of CD19^+^CD24^hi^CD38^hi^ cells with the glomerular filtration rate as calculated by MDRD (eGFR) was performed with Pearson’s test after proving normal distribution of the data. A *p* value < 0.05 was considered as statistically significant.

## 3 Results

### Calcineurin inhibitors reduce the percentages of both CD19^+^CD24^hi^CD38^hi^ Bregs and IL-10 producing Bregs *in vivo* and *in vitro*

CD24^hi^CD38^hi^ B-cells were assessed in renal transplant recipients receiving an immunosuppressive therapy consisting of prednisone, a mycophenol acid derivative and CsA (n = 11) or tacrolimus (n = 35) or age- and gender-matched healthy subjects (n = 16). Demographic data and clinical chemistry is shown in Table 1. The percentages of peripheral circulating CD19^+^ B-cells among lymphocytes was reduced both in TAC (6.4 ±0.7%) and CsA (3.6 ±0.7%) treated patients compared to healthy subjects (9.1 ±0.6%, Fig 1C). Repeatidly, the vast majority of IL-10 producing B-cells were found to express CD38 in a low or intermediate degree (Fig 1A, right panel) and only ~14.5% of the CD24^hi^CD38^hi^ expressing cells were IL-10 positive in healthy subjects (n = 9), transplanted patients (n = 9) and healthy subjects receiving CsA (n = 4; not shown). Among B-cells, the distinct subset of CD24^hi^CD38^hi^ cells was found to be 4.7 ±0.5% in healthy subjects, 1.4 ±0.4% in tacrolimus treated patients and almost blunted in patients receiving CsA (1.1 ±0.5%) (Fig 1B and 1D). There was neither a correlation in between the amount of CD19^+^CD24^hi^CD38^hi^ B-cells and the timepoint of sample assessment (Fig 1E) or the trough levels of tacrolimus (r = -0.18 p = 0.3) or CsA (r = 0.48 p = 0.18, not shown), respectively.

**Table 1:** Patient data.

		HS	TAC	CsA
		(n = 16)	(n = 35)	(n = 11)
**Age (yrs)**		44 ±3	49 ±11	57 ±12
**Gender (m/f)**		1.3: 1	1.2: 1	1.75: 1
**Donor (LDR vs CDR)**		-	8 vs. 27	4 vs. 7
	**AAMR**	-	-	-
**Renal allograft Rejection**	**CAMR**	-	3	-
	**ACMR**	-	6	1
**Analysis post Tx (in months)**		-	24 ±26	93 ±54
**Creatinin (mg/dL)**		-	1.8 ±0.7	2.1 ±1.1
**MDRD (mL/min)**		-	44.1 ±13.4	34.8 ±12.0
**Trough level (ng/ml)**		-	6.2 ±2.7	106 ±30

AAMR, Acute antibody mediated rejection; CAMR, Chronic antibody mediated rejection; ACMR, Acute cellular mediated rejection, CCMR, Chronic cellular mediated rejection

### The percentages of peripherally circulating IL-10 producing B-cells is reduced in calcineurin inhibitor treated renal transplant recipients

**Fig 2:**
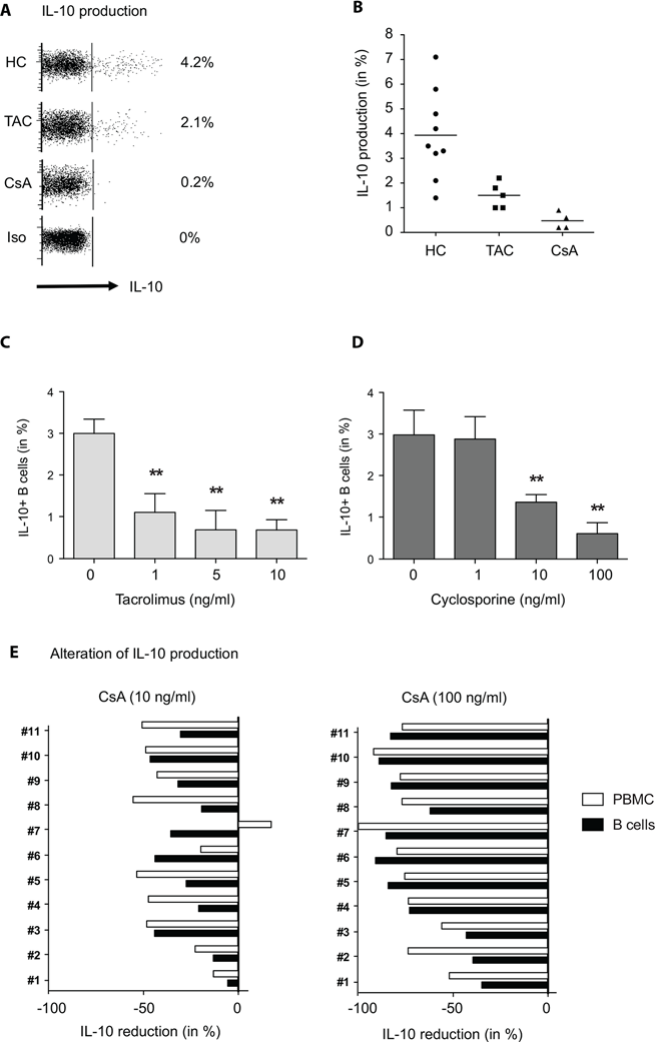
The calcineurin inhibitors tacrolimus and CsA inhibit IL-10 expression of B-cells *in vitro* and *in vivo*. Freshly isolated PBMCs from healthy subjects (n = 9) and renal transplant recipients (n = 9) were mitogen/toll-like-receptor 9 stimulated for 72 hours and subsequently stained for surface CD19 and intracellular IL-10 or with the respective isotype control antibodies **(A).** The representative dot plots on the upper left in **A** show the intracellular IL-10 expression of stimulated CD19^+^ B-cells from a healthy subject and representative renal transplant recipients receiving either tacrolimus or CsA (after gating on CD19^+^ B-cells). The scatter plot in **B** summarizes the results of multiple unrelated experiments. PBMCs of healthy subjects were stimulated like described before in presence or absence of different concentrations of tacrolimus (n = 4) **(C)** or CsA (n = 4) **(D)**, as indicated. The bar graphs in **E** depict the decline of IL-10 production (in %) of PBMCs vs. positively isolated CD19^+^ B-cells after stimulation co-cultured with CsA (n = 9 healthy subjects). 7-AAD staining was performed to ensure viability of cell culture.

After *ex vivo* mitogen stimulation of PBMCs containing the whole white blood cell population with a TLR-9 agonist, ∼4% of all CD19^+^ B-cells showed IL-10 production in healthy subjects (n = 9) and even fewer in TAC (n = 5) or CsA (n = 4) treated patients (1.5 ±0.5% and 0.5 ±0.3%, respectively) (Fig 2A–2D). None of these patients shown in Fig 2 did experience allograft rejection events before or after measurement, they had a stable renal function (mean eGFR 47.3 ±10.2ml/min) and the mean time after transplantation was 65 ±32 months. All patients were treated with steroids and MMF in addition to the CNIs. Co-culture of mitogen stimulated PBMCs of healthy subjects (n = 4) in presence of increasing concentrations of CsA or TAC resulted in a dose dependent reduction of IL-10 production after three days of incubation. Interestingly, the drug concentrations needed to suppress IL-10 production in B-cells were comparable to the trough levels applied in the clinical setting of renal transplantation (Fig 2A–2D). To get more insight in the possible mechanism and elucidate whether this effect was a secondary consequence of an IL-2 deprivation and abortion of T-cell mediated activation of Bregs, both PBMCs as well as positively isolated B-cells of nine healthy subjects were stimulated and co-cultured with CsA as previously performed. In both cell suspensions IL-10 production was significantly reduced compared to CsA free cultures (Fig 2E). The data from these unrelated experiments provided evidence that CsA directly inhibited B-cells to produce IL-10.

### CsA reduced the percentage of CD19^+^CD24^hi^CD38^hi^ cells and IL-10^+^ B-cells in healthy non-transplanted subjects

**Fig 3:**
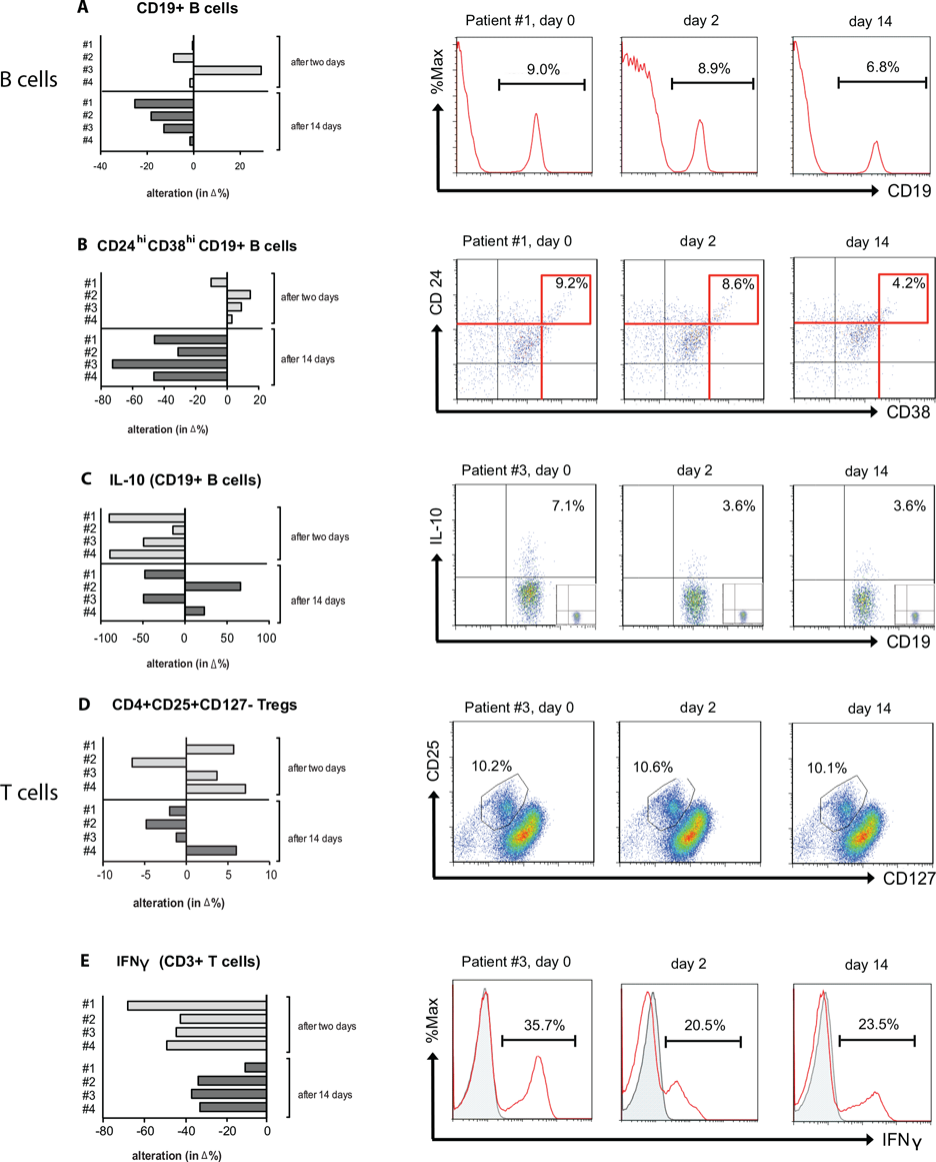
CsA reduced both the percentage of CD19^+^CD24^hi^CD38^hi^ B-cells and IL-10^+^ B-cells in healthy subjects. Healthy subjects (n = 4) were treated with CsA for 14 days, two days receiving a high dosage followed by an additional 12 days with a low dosage treatment. The bargraphs in panels **A-C** show the decline of peripheral circulating CD19^+^CD24^hi^CD38^hi^ B-cells and their intracellular production of IL-10^+^ compared to day 0 (in %) while the percentage of CD4^+^CD25^+^CD127^-^ Tregs was unaltered during CsA treatment **(D)**. The intracellular IFN-γ production of CD3^+^ T-cells was assessed and served as a positive control reaction **(E)**. Corresponding representative dotplots or histograms are displayed on the right **(A-E)**.

To confirm that the decrease of both B-cells and Bregs in the peripheral circulation is directly caused by calcineurin inhibition, we took advantage of a pre-existing study in which healthy subjects were given CsA in clinical dosages. Whole blood specimen of four healthy subjects were analyzed for the percentage of CD19^+^CD24^hi^CD38^hi^ and CD19^+^ B-cells. In addition, the amount of IL-10 producing B-cells was assessed using isolated PBMCs and culturing them as described above, before and after a 14 days lasting treatment with CsA. CsA was given body weight adapted 4 times during the first two days using 2.5 mg/kg (high dosage) and then reduced to 0.25 mg/kg (Fig 3). After the first two days the mean drug trough level was 117±29 ng/mL. The frequency of CD19^+^ B-cells including the CD24^hi^CD38^hi^ subset was unaltered (Fig 3A and 3B). In contrast, the production of IL-10 in B-cells decreased from 3.7 ±1.3% to 1.5 ±0.8% and IFN-γ in CD3^+^ T-cells from 36.2 ±0.5% to 15.6 ±2.5% at day 2. After additional 12 days of low dose CsA treatment all analyzed parameters did further substantially decrease: CD19^+^ B-cells from 8.2 ±0.6% at day 0 to 6.9 ±0.3% after 14 days of treatment (Fig 3A), CD24^hi^ CD38^hi^ B-cells from 4.9 ±1.3% to 2.7 ±0.9% (Fig 3B), IL-10^+^ B-cells from 3.7 ±1.3% to 2.7 ±0.5% (Fig 3C), and IFN-γ producing lymphocytes from 36.2 ±0.5% to 26.1 ±2.5% (Fig 3D). Only the frequency of CD25^hi^CD127^lo^ expressing CD4+ Tregs remained unaltered. Altogether, these results demonstrate that calcineurin inhibitors reduced the production of IL10^+^ in B-cells and that this effect is not restricted to a solid organ transplant setting or a co-medication with steroids or MPA.

### A low percentage of CD19^+^CD24^hi^CD38^hi^ Bregs in the peripheral blood of renal transplant recipients is associated with a reduced glomerular filtration rate

**Fig 4:**
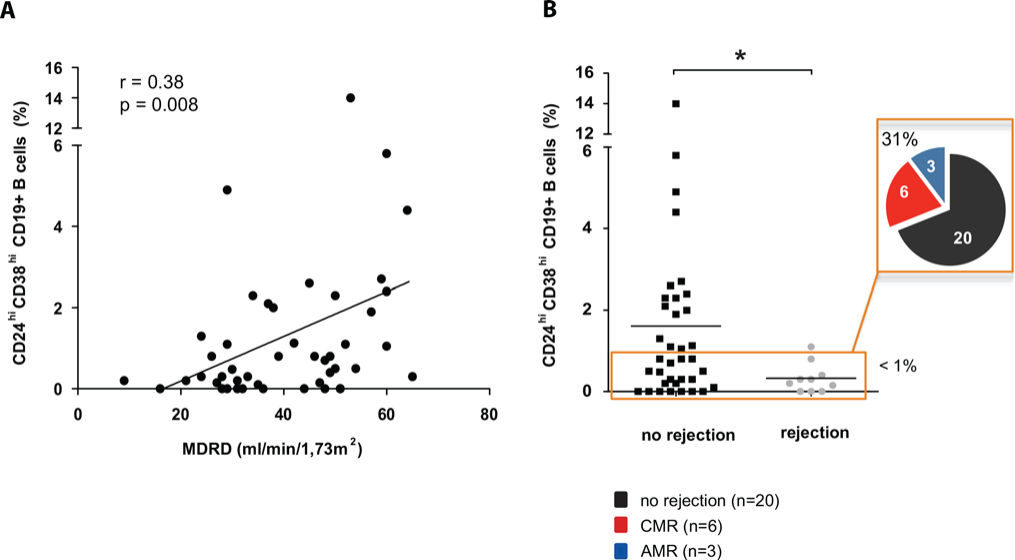
The amount of peripheral circulating CD19^+^CD24^hi^CD38^hi^ cells correlated with the clinical outcome after kidney transplantation A low amount of peripheral circulating CD19^+^CD24^hi^CD38^hi^ cells was associated with a lower eGFR **(A).** The orange-framed box in **B** indicates a subgroup of 29 patients that exhibited <1% of CD24^hi^38^hi^ expressing CD19^+^ peripherally circulating B-cells. 31% (n = 9) of these patients experienced a biopsy proven rejection event 24 months before or after the analyses (5 patients before and 4 patients within 24 months after sample assessment).

To evaluate the relevance of our findings the correlation between the percentage of peripherally circulating CD19^+^CD24^hi^CD38^hi^ B-cells and renal allograft function (MDRD formula, eGFR) was tested (Spearman`s rank correlation test, Fig 4A). In our cohort of 46 patients (overall mean eGFR 40.6 ±13.4), a high amount of CD19^+^CD24^hi^CD38^hi^ cells was associated with a higher eGFR (r = 0.38, p = 0.0079). When CsA and TAC treated patients were analysed separately, this correlation was mainly observed in the cohort of tacrolimus treated patients (TAC, n = 35; r = 0.46 p = 0.006; CsA, n = 11 r = 0.07 p = 0.81; not shown). Moreover, patients with a low amount of peripherally circulating CD19^+^CD24^hi^CD38^hi^ B-cells show an elevated frequency of biopsy proven allograft rejection events (Fig 4B, *p*<0.05), although this analysis did not reach statistical significance using the Fisher’s exact test (*p*<0.07). The likelihood ratio to develop an allograft rejection with a CD19^+^CD24^hi^CD38^hi^ B-cell frequency < 1% was 1.6.

Among 29 patients with less than 1% of CD19^+^CD24^hi^CD38^hi^ cells, 9 cases (31%) displayed a biopsy proven allograft rejection. Of these 9, five patients developed a rejection within 24 months before sample assessment, four of them were of acute cellular (ACMR) and one of chronic humoral origin (CAMR). Four patients (1 ACMR, 1 CCMR, 2 CAMR) developed allograft rejection within 24 months after measurement displaying no signs of a loss in renal allograft function at the time point of sample assessment. In contrast, only one case of acute rejection (6%, ACMR, BANFF 1A) occurred in the remaining group of 17 patients with greater than 1% of peripherally circulating CD19^+^CD24^hi^CD38^hi^ B-cells (assessed 2 months before the rejection event, Fig 4B). Prior to Tx, none of the patients displayed anti-HLA donor specific antibodies (DSA). During follow up, one patient developed de novo donor-specific anti-HLA antibodies and suffered from a humoral rejection (CAMR). The majority of biopsy proven acute rejections in this study was cellular mediated (ACMR/CCMR, 6/1), only three were of an antibody mediated origin (AAMR/CAMR, 1/2; Table 1). Reduced frequencies of transitional CD19^+^CD24^hi^CD38^hi^ B-cells were associated with a higher risk of developing an allograft rejection but only 30% of these cases were antibody mediated (AAMR or ACMR).

Statistical analysis of the amount of CD19^+^CD24^hi^CD38^hi^ B-cells and TAC (r = 0.03, p = 0.83) or CsA (r = 0.48, p = 0.18) trough levels (Fig 4C and 4D), the time after transplantation, the dosage of co-medication (steroids and or MPA), CMV infections or the age of allograft recipients or donors revealed no significant correlation (not shown).

## 4 Discussion

The presence and function of B regulatory cells are of increasing interest in solid organ transplantation. In particular, the immature transitional CD19^+^ B-cell phenotype characterized by the surface expression of CD24^hi^CD38^hi^ was previously suggested to play an important role not only in maintaining long-term allograft function but also in promoting allograft tolerance [21–23].

In this study, we found a reduced amount of peripheral circulating CD19^+^CD24^hi^CD38^hi^ B-cells in renal transplant recipients receiving a calcineurin inhibitor based immunosuppressive therapy when compared to healthy subjects. A low percentage of peripheral circulating CD19^+^CD24^hi^CD38^hi^ B-cells in these patients was associated with a worse clinical outcome in allograft function (eGFR) and allograft rejection events. *In vivo* studies involving CsA treated healthy subjects proved that the reduced surface expression of the CD24 and CD38 molecules as well as the impaired IL-10 production was caused by calcineurin inhibition. Further *in vitro* experiments using immunomagnetic isolated B-cells confirmed that CsA directly affects the Breg’s IL-10 production and excluded the possibility of IL-2 deprivation and/or T helper cell mediated activation of Bregs.

The most widely studied mechanism by which Bregs regulate immune responses is via direct suppression of CD4^+^ T-cells through provision of IL-10. The CD19^+^CD24^hi^CD38^hi^ immature B-cell subset investigated in this study was also shown to exhibit regulatory capability mainly through the release of IL-10 [14]. However, as revealed in patients with SLE, their suppressive capacity was also dependent on CD80/CD86 expression indicating an important role for contact-mediated suppression apart from their production of IL-10 [14]. Although not a primary aim of this study, a further interesting observation was that only a minority of ~14.5% among CD19^+^CD24^hi^CD38^hi^ cells was found to produce IL-10 suggesting indeed that these transitional, immature B-cells are heterogenous and employ alternative mechanisms to exert their regulatory function independently of IL-10. Whether this mechanism is based on secretion of further suppressive soluble factors or solely vial cell-cell contact is currently under investigation.

Not only functional defects but also a numerical reduction has been described for CD19^+^CD24^hi^CD38^hi^ immature B-cells and IL-10 producing Breg cells in autoimmune diseases, including SLE and multiple sclerosis [8]. In this study, both the percentage of CD19^+^CD24^hi^CD38^hi^ immature B-cells in the peripheral circulation as well as the ability of B-cells to produce IL-10 were affected by calcineurin inhibition *in vivo* and *in vitro*. Similar findings were described in a very recent work by Shabir et al., who demonstrated a significant decrease of CD19^+^CD24^hi^CD38^hi^ cells in pre- to three months posttransplant specimen and normalizing again six months after transplantation [24]. Recently, Chung et al. [25] also reported a decrease of the CD19+CD24^hi^CD38^hi^ immature B-cell subset and their production of IL-10 in a cohort of 21 renal transplant patients undergoing a tacrolimus based immunosuppression. Although the authors performed their assay only shortly before and until one month after transplantation these results corroborate our observations. The primary target of CNIs is to prevent the activation of NFAT in T-cells leading to the suppression of IL2-gene transcription. Therefore, a reduced number of T-cells available for B-cell help caused by CNI mediated inhibition of T-cell proliferation or the induction of T-cell apoptosis may serve as an obvious and appealing explanation. However, studies elucidating direct effects of CNIs on B-cells are scarce. Especially, their effect on Breg subsets has not been elucidated so far. In purified human B-cells, Heidt et al. and Traitanon et al. demonstrated that in contrast to CsA and tacrolimus, Rapamycin and MPA profoundly inhibited B-cell proliferation [26], maturation into plasma cells [27] and immunoglobulin production [26], respectively. In addition, the *in vitro* effects of cyclosporine and tacrolimus on IL-6 and IL-10 production were less pronounced and depended on dosage and type of stimulation [26, 27]. Our *in vivo* findings in healthy subjects and in CsA co-cultured immunomagnetic negatively isolated B-cells provide new biologically relevant aspects on this issue, because they clearly demonstrate that the production of IL-10 and hence the regulatory function of Bregs seem to be directly hampered by calcineurin inhibition. These observations furthermore reveal that the IL-10 production of Breg cells may occur without any T-cell interaction. However, the exact mechanism by which Calcineurin inhibitors suppress Breg function remains to be further elucidated.

Although a dose dependent reduction of IL-10 production was observed in our *in vitro* experiments, a correlation of drug trough levels with the amount of CD19^+^CD24^hi^CD38^hi^ Bregs in the renal transplant setting was not found. The reason may be a longer exposition of B-cells in patients receiving calcineurin inhibitors one the one hand or the polypharmaceutical treatment on the other. Indeed, most results in renal transplant studies are affected by a multicompound immunosuppressive therapy regime [28] and the majority of the CNI treated recipients receive co-medications with corticosteroids and/or MPA. This makes it difficult to dissect which drug in fact is responsible for the observed reduction of Breg cells. To solve this issue, a subcohort of non-transplanted healthy subjects that received a mono-therapy with CsA was included in this study. To the best of our knowledge this is a unique and so far unreported experiment. It enabled us to demonstrate that the observed reduction in CD19^+^CD24^hi^CD38^hi^ immature Breg subsets is solely mediated by Calcineurin inhibition and that this is independent of a renal transplant setting, renal allograft function or a co-medication with steroids or MPA derivatives.

Breg cells seem to play a crucial role in balancing the alloreactive immune response, as kidney transplanted patients with <1% of CD19^+^CD24^hi^CD38^hi^ B-cells had a significant higher risk of experiencing allograft rejection events. Patients that suffered from rejection before or after sample assessment had a significant lower frequency of CD19^+^CD24^hi^CD38^hi^ B-cells. Support to these findings is provided by Chesneau and coworkers who demonstrated that tolerant human renal transplant recipients exhibited higher frequencies of CD20^+^CD24^hi^CD38^hi^ transitional and CD20^+^CD38^lo^CD24^lo^ naive B-cells compared to patients with stable allograft function under immunosuppressive treatment [23]. Moreover, Silva et al. showed that operational tolerant subjects exhibited preservation in the number of circulating B-cells, particularly for the immature CD19^+^CD24^hi^CD38^hi^ Bregs, in contrast to decreased numbers in subjects with chronic rejection [29]. Most recently, Borrows' group also described a protective function of transitional CD19^+^CD24^hi^CD38^hi^ B-cells preventing allograft rejection within the first year of kidney transplantation [24]. Our findings are in line with Shabir et al. [24] who also demonstrated that in a cohort of 73 *de novo* kidney transplanted patients a low amount of transitional B-cells correlated with a higher risk of allograft rejection.

Interestingly, allograft rejections in patients exhibiting <1% of peripherally circulating CD19^+^CD24^hi^CD38^hi^ Bregs were mainly cellular mediated (ACMR). Only 30% of these cases were antibody mediated (CAMR) as might be expected (Table 1, Fig 4). It is a limitation of this study that the development of *de novo* DSA was not assessed in a time-dependent fashion during our routine follow-up, since this might have enabled us to investigate the relationship in between DSA-producing and tolerogenic B-cells. Irrespectively, these results still allow the conclusion that a conserved Breg subset may protect rather from cellular than from humoral allograft rejections. Further multicenter studies including larger patient cohorts will be needed to fully elucidate these preliminary findings as monitoring of B-cell phenotypes could be of importance to both analyze and better predict the characteristics of a cellular or humoral alloimmune response.

Altogether, these study results suggest that subsets of the CD19^+^CD24^hi^CD38^hi^ Breg population and possibly their production of IL-10 may be an important regulator of the inflammatory alloimmune response and that deficiency or loss of these transitional immature Bregs might be involved in the process of acute and chronic allograft rejection. Moreover, we observed a so far underestimated side effect of CNI inhibitors, especially of CsA in reducing the frequency and hampering the function of immature transitional Bregs that may compromise renal allograft function in the longterm. Whether the impairment of the transitional immature B-cell subset can be considered as an individual predisposition to a toxic side effect of Calcineurin inhibition needs to be clarified in further studies co-investigating pharmacodynamic drug effects.

The analyses of CD19^+^CD24^hi^CD38^hi^ Bregs in this study provide a further supplemental piece of relevant information on B regulatory cell components of the recipient’s immune system after renal transplantation. Patients displaying higher transitional B-cell frequencies experienced reduced rejection rates. In addition, CNI treatment results in a reduction of transitional B-cells and hence may negatively influence the development of a favorably balanced B-cell compartment. To investigate whether renal patients are at risk to develop allograft rejection events it thus may be helpful to determine the amount of CD19^+^CD24^hi^CD38^hi^ Bregs periodically. The monitoring of CD19^+^CD24^hi^CD38^hi^ Bregs may thus have the potential to serve as an adjunct tool to improve the monitoring of the subjects peripheral cellular immunity in order to prevent renal allograft rejection independent from determining drug trough levels. Given the fact that drug trough levels provide no insight in the subjects pharmacodynamic response towards CNIs, a regular assessment of the numerical composition and function of the peripheral cellular immune regulation may gain in importance in the near future [30]. Further studies with a longitudinal monitoring of patients will therefore be necessary to define cut-off values of peripheral circulating CD19^+^CD24^hi^CD38^hi^ Bregs cells that may predict allograft rejection events after solid organ transplantation.

## Abbreviations

AAMR: Acute Antibody Mediated Rejection
ACMR: Acute Cellular Mediated Rejection
Breg: regulatory B-Lymphocytes
CCMR: Chronic cellular mediated rejection
CD: cadaveric donation
CNI: Calcineurin Inhibitor
CsA: Cyclosporine A
DSA: donor-specific allo-antibodies
eGFR: estimated Glomerular Filtration Rate
FACS: Fluorescence Activated Cell Sorting
HS: healthy subjects
IL: Interleukin
LRD: living related donation
MPA: mycophenolate mofetil
PBMC: Peripheral Blood Mononuclear Cells
RTx: renal transplantation
TAC: tacrolimus
Th: T helper cell
Tx: transplantation

## References

[1] Lund FE, Randall TD. Effector and regulatory B cells: modulators of CD4+ T cell immunity. Nat Rev Immunol. 2010;10(4):236–47. 10.1038/nri2729 20224569 PMC3038334

[2] Kalampokis I, Yoshizaki A, Tedder TF. IL-10-producing regulatory B cells (B10 cells) in autoimmune disease. Arthritis Res Ther. 2013;15 Suppl 1:S1 10.1186/ar3907 23566714 PMC3624502

[3] Nankivell BJ, Alexander SI. Rejection of the kidney allograft. N Engl J Med. 2010;363(15):1451–62. 10.1056/NEJMra0902927 . 20925547

[4] Loupy A, Hill GS, Jordan SC. The impact of donor-specific anti-HLA antibodies on late kidney allograft failure. Nat Rev Nephrol. 2012;8(6):348–57. 10.1038/nrneph.2012.81 . 22508180

[5] Shen P, Roch T, Lampropoulou V, O'Connor RA, Stervbo U, Hilgenberg E, et al IL-35-producing B cells are critical regulators of immunity during autoimmune and infectious diseases. Nature. 2014;507(7492):366–70. 10.1038/nature12979 . 24572363 PMC4260166

[6] Fillatreau S. Cytokine-producing B cells as regulators of pathogenic and protective immune responses. Ann Rheum Dis. 2013;72 Suppl 2:ii80–4. Epub 2012/12/21. 10.1136/annrheumdis-2012-202253 . 23253921

[7] Ray A, Basu S, Williams CB, Salzman NH, Dittel BN. A novel IL-10-independent regulatory role for B cells in suppressing autoimmunity by maintenance of regulatory T cells via GITR ligand. J Immunol. 2012;188(7):3188–98. 10.4049/jimmunol.1103354 22368274 PMC3311743

[8] Mauri C, Bosma A. Immune regulatory function of B cells. Annu Rev Immunol. 2012;30:221–41. Epub 2012/01/10. 10.1146/annurev-immunol-020711-074934 . 22224776

[9] Rosser EC, Mauri C. Regulatory B cells: origin, phenotype, and function. Immunity. 2015;42(4):607–12. 10.1016/j.immuni.2015.04.005 . 25902480

[10] Yanaba K, Bouaziz JD, Haas KM, Poe JC, Fujimoto M, Tedder TF. A regulatory B cell subset with a unique CD1dhiCD5+ phenotype controls T cell-dependent inflammatory responses. Immunity. 2008;28(5):639–50. Epub 2008/05/17. 10.1016/j.immuni.2008.03.017 . 18482568

[11] Ding Q, Yeung M, Camirand G, Zeng Q, Akiba H, Yagita H, et al Regulatory B cells are identified by expression of TIM-1 and can be induced through TIM-1 ligation to promote tolerance in mice. J Clin Invest. 2011;121(9):3645–56. 10.1172/JCI46274 21821911 PMC3163958

[12] Xiao S, Brooks CR, Zhu C, Wu C, Sweere JM, Petecka S, et al Defect in regulatory B-cell function and development of systemic autoimmunity in T-cell Ig mucin 1 (Tim-1) mucin domain-mutant mice. Proc Natl Acad Sci U S A. 2012;109(30):12105–10. 10.1073/pnas.1120914109 22773818 PMC3409739

[13] Braza F, Chesne J, Castagnet S, Magnan A, Brouard S. Regulatory functions of B cells in allergic diseases. Allergy. 2014;69(11):1454–63. 10.1111/all.12490 . 25060230

[14] Blair PA, Norena LY, Flores-Borja F, Rawlings DJ, Isenberg DA, Ehrenstein MR, et al CD19(+)CD24(hi)CD38(hi) B cells exhibit regulatory capacity in healthy individuals but are functionally impaired in systemic Lupus Erythematosus patients. Immunity. 2010;32(1):129–40. Epub 2010/01/19. 10.1016/j.immuni.2009.11.009 . 20079667

[15] Wilde B, Thewissen M, Damoiseaux J, Knippenberg S, Hilhorst M, van Paassen P, et al Regulatory B cells in ANCA-associated vasculitis. Ann Rheum Dis. 2013;72(8):1416–9. 10.1136/annrheumdis-2012-202986 . 23666929

[16] Nouel A, Simon Q, Jamin C, Pers JO, Hillion S. Regulatory B cells: an exciting target for future therapeutics in transplantation. Front Immunol. 2014;5:11 10.3389/fimmu.2014.00011 24478776 PMC3897876

[17] Duddy M, Niino M, Adatia F, Hebert S, Freedman M, Atkins H, et al Distinct effector cytokine profiles of memory and naive human B cell subsets and implication in multiple sclerosis. J Immunol. 2007;178(10):6092–9. . 17475834 10.4049/jimmunol.178.10.6092

[18] Jagannathan M, McDonnell M, Liang Y, Hasturk H, Hetzel J, Rubin D, et al Toll-like receptors regulate B cell cytokine production in patients with diabetes. Diabetologia. 2010;53(7):1461–71. 10.1007/s00125-010-1730-z 20383694 PMC2895399

[19] Iwata Y, Matsushita T, Horikawa M, Dilillo DJ, Yanaba K, Venturi GM, et al Characterization of a rare IL-10-competent B-cell subset in humans that parallels mouse regulatory B10 cells. Blood. 2011;117(2):530–41. Epub 2010/10/22. 10.1182/blood-2010-07-294249 20962324 PMC3031478

[20] Chesneau M, Michel L, Degauque N, Brouard S. Regulatory B Cells and Tolerance in Transplantation: From Animal Models to Human. Front Immunol. 2013;4:497 Epub 2014/01/16. 10.3389/fimmu.2013.00497 24427159 PMC3876023

[21] Newell KA, Asare A, Kirk AD, Gisler TD, Bourcier K, Suthanthiran M, et al Identification of a B cell signature associated with renal transplant tolerance in humans. J Clin Invest. 2010;120(6):1836–47. Epub 2010/05/27. 10.1172/JCI39933 20501946 PMC2877933

[22] Sagoo P, Perucha E, Sawitzki B, Tomiuk S, Stephens DA, Miqueu P, et al Development of a cross-platform biomarker signature to detect renal transplant tolerance in humans. J Clin Invest. 2010;120(6):1848–61. 10.1172/JCI39922 20501943 PMC2877932

[23] Chesneau M, Pallier A, Braza F, Lacombe G, Le Gallou S, Baron D, et al Unique B cell differentiation profile in tolerant kidney transplant patients. Am J Transplant. 2014;14(1):144–55. Epub 2013/12/21. 10.1111/ajt.12508 . 24354874

[24] Shabir S, Girdlestone J, Briggs D, Kaul B, Smith H, Daga S, et al Transitional B lymphocytes are associated with protection from kidney allograft rejection: a prospective study. Am J Transplant. 2015;15(5):1384–91. 10.1111/ajt.13122 . 25808898

[25] Chung BH, Kim KW, Yu JH, Kim BM, Choi BS, Park CW, et al Decrease of immature B cell and interleukin-10 during early-post-transplant period in renal transplant recipients under tacrolimus based immunosuppression. Transpl Immunol. 2014;30(4):159–67. 10.1016/j.trim.2014.03.003 . 24709525

[26] Heidt S, Roelen DL, Eijsink C, van Kooten C, Claas FH, Mulder A. Effects of immunosuppressive drugs on purified human B cells: evidence supporting the use of MMF and rapamycin. Transplantation. 2008;86(9):1292–300. 10.1097/TP.0b013e3181874a36 . 19005412

[27] Traitanon O, Mathew JM, La Monica G, Xu L, Mas V, Gallon L. Differential Effects of Tacrolimus versus Sirolimus on the Proliferation, Activation and Differentiation of Human B Cells. PLoS One. 2015;10(6):e0129658 10.1371/journal.pone.0129658 26087255 PMC4472515

[28] Hoerning A, Kohler S, Jun C, Lu J, Fu J, Tebbe B, et al Cyclosporin but not everolimus inhibits chemokine receptor expression on CD4+ T cell subsets circulating in the peripheral blood of renal transplant recipients. Clinical and experimental immunology. 2012;168(2):251–9. Epub 2012/04/05. 10.1111/j.1365-2249.2012.04571.x 22471287 PMC3390527

[29] Silva HM, Takenaka MC, Moraes-Vieira PM, Monteiro SM, Hernandez MO, Chaara W, et al Preserving the B-cell compartment favors operational tolerance in human renal transplantation. Molecular medicine. 2012;18:733–43. Epub 2012/01/19. 10.2119/molmed.2011.00281 22252714 PMC3409285

[30] Albring A, Wendt L, Harz N, Engler H, Wilde B, Kribben A, et al Relationship between pharmacokinetics and pharmacodynamics of calcineurin inhibitors in renal transplant patients. Clin Transplant. 2015;29(4):294–300. 10.1111/ctr.12504 . 25557538

